# Pharmacokinetic Characteristics of Tolfenamic Acid in Freshwater Crocodiles (*Crocodylus siamensis*)

**DOI:** 10.3390/ani15050684

**Published:** 2025-02-26

**Authors:** Seavchou Laut, Saranya Poapolathep, Kraisiri Khidkhan, Narumol Klangkaew, Napasorn Phaochoosak, Tara Wongwaipairoj, Mario Giorgi, Elisa Escudero, Pedro Marin, Amnart Poapolathep

**Affiliations:** 1 Department of Pharmacology, Faculty of Veterinary Medicine, Kasetsart University, Bangkok 10900, Thailand; seavchou.l@ku.th (S.L.); fvetsys@ku.ac.th (S.P.); kraisiri.kh@ku.th (K.K.); fvetnak@ku.ac.th (N.K.); fvetanp@ku.ac.th (N.P.); 2 Wongveerakit Crocodile Farm, Bo Phloi, Kanchanaburi 71160, Thailand; tara.wongwai@gmail.com; 3 Department of Veterinary Science, University of Pisa, 56112 Pisa, Italy; mario.giorgi@unipi.it; 4 Department of Pharmacology, Faculty of Veterinary Medicine, University of Murcia, 30100 Murcia, Spain; escudero@um.es

**Keywords:** freshwater crocodile, tolfenamic acid, plasma, pharmacokinetics, NSAIDs, HPLC

## Abstract

The present study was undertaken to characterize the plasma kinetic disposition of tolfenamic acid (TA) in freshwater crocodiles. In total, 15 freshwater crocodiles were used in the experiment and randomly divided into three groups, with TA administered at 2 mg/kg body weight (b.w.) intravenously (IV) or at 2 or 4 mg/kg b.w. intramuscularly (IM). Blood samples were collected at predetermined times up to 168 h after IV or IM drug administration. Plasma concentrations of TA were determined using validated high-performance liquid chromatography with a UV detector and then analyzed based on the non-compartmental method. The maximum concentration values of TA were 3.03 µg/mL and 6.83 µg/mL following IM administration at a dose of 2 mg/kg b.w. or 4 mg/kg b.w., respectively. The elimination half-lives were 21.89 h (2 mg/kg; IV), 17.74 h (2 mg/kg; IM), and 13.57 h (4 mg/kg; IM). Following IV administration, the volume of distribution and clearance were 1.58 L/kg and 50.04 mL/h/kg, respectively. The absolute IM bioavailability was 71.0% at a dose of 2 mg/kg b.w. and 92.63% at a dose of 4 mg/kg b.w. The average ± SD of plasma protein binding of TA was 26.15 ± 4.93%. Good bioavailability levels and favorable plasma concentrations of TA were obtained in freshwater crocodiles after IM administrations, considering that this is the preferred route of drug administration in freshwater crocodiles. Multi-dose and pharmacodynamic studies are needed to better establish the safety and efficacy of using TA in this crocodilian species.

## Simple Summary

Reptile analgesia data for pain management are not well-documented, with an even greater gap in knowledge for freshwater crocodiles, where drug dosing is commonly inferred from doses reported for other animal species. It is necessary to gain a thorough understanding of the disposition of nonsteroidal anti-inflammatory agents in reptiles and to assess their potential toxicity before any recommendations can be made. No studies have yet been carried out to investigate the pharmacokinetics of tolfenamic acid (TA) in freshwater crocodiles. Therefore, the objective of this study was to investigate the disposition kinetics of TA in freshwater crocodiles. Based on the results, intravascular or intramuscular administration of TA was well-tolerated, with no adverse effects. Thus, favorable pharmacokinetic characteristics were suggested for therapeutic use.

## 1. Introduction

Detecting pain in reptiles can be challenging, but it is reasonable to assume that conditions that cause pain in mammals would produce similar pain responses in reptiles [1,2]. Various harmful stimuli can lead to inflammatory pain, caused thermally (such as from burns), mechanically (such as injuries from other animals), and chemically (such as proinflammatory mediators due to infections) [3]. Untreated pain can affect the inflammatory process, tissue healing, and immune function [4]. Therefore, the use of anesthetic and anti-inflammatory drugs is necessary to control pain and inflammation in reptiles. Nonetheless, it is important to recognize that pain can manifest in various ways across different animal species. Consequently, caution in generalized applications is warranted; specifically, the administration of analgesics in reptiles should be carefully considered. Clinicians are encouraged to exercise their best judgment and to assess each case individually, while also consulting the latest literature for current information [5]. However, pharmacokinetic and pharmacodynamic studies with these drugs are very scarce in crocodilians, making it difficult to establish adequate doses and dosing regimens with a minimal risk of adverse effects [1].

Tolfenamic acid (TA), with the molecular structure N-(2-methly-3-chlorophenly), is an anthranilic acid and benzoic acid derivative that is classified as a nonsteroidal anti-inflammatory agent (NSAID) [6]. TA belongs to the fenamate group and has analgesic, antipyretic, and anti-inflammatory properties [7]. It works by inhibiting cyclooxygenase (COX) 1 and COX 2, leading to a reduction in the production of prostaglandins and thromboxanes [8]. This inhibition of prostaglandin synthesis is responsible for the drug’s therapeutic effects. In addition, thromboxane A2 synthesis is suppressed by thromboxane synthase, which decreases platelet aggregation. The European Medicines Agency (EMA) has approved the use of TA to treat mastitis and respiratory infections in cattle and goats, to treat metritis-mastitis agalactia in pigs, and to provide postoperative analgesia in cats and dogs [6,9]. Studies on the pharmacokinetics of TA have been explored in various animal species, covering mammals, avians, reptiles and fish, such as: goats, sheep, geese, calves, and rats [10,11,12,13,14]; green sea turtles, red-eared sliders, and hawksbill turtles [15,16,17]; Japanese quails and ducks [18,19]; and rainbow trout [20]. To date, there have been no reported studies of TA in freshwater crocodiles. Despite the widespread use of NSAIDs in veterinary medicine, no studies have reported the pharmacokinetics of NSAIDs in crocodiles. Freshwater crocodiles are subjected to injuries, surgical procedures, and inflammatory conditions in both wild and captive settings. The lack of research on TA in freshwater crocodiles limits veterinary treatment options. Understanding its pharmacokinetics is essential for determining its potential as an effective NSAID for crocodilian medicine. Tolfenamic acid (TA) was selected because of its potential safety and efficacy advantages which have been studied in a variety of species, including mammals, birds, reptiles, and fish. TA has shown a longer half-life in certain species, which could reduce the need for frequent dosing. This is particularly beneficial in crocodiles because frequent handling can induce stress and risk injury. Additionally, TA is widely available and cost-effective, making it a practical option for veterinary use. Therefore, the current study was conducted to examine the pharmacokinetic characteristics of TA following intramuscular (IM) administration at 2 or 4 mg/kg b.w. or intravenous (IV) administration at 2 mg/kg b.w. in freshwater crocodiles (*Crocodylus siamensis*).

## 2. Materials and Methods

### 2.1. Animals

A sample of 15 healthy and clinically normal freshwater crocodiles (*Crocodylus siamensis*) were assessed based on clinical examination and a complete blood count. The freshwater crocodiles were aged 2.5–3.2 years with a mean ± SD body weight of 7.52 ± 0.94 kg, and body lengths in the range of 119–146 cm. The protocol conducted in the study strictly followed the guidelines for the use of animals and was approved by the Animal Ethics Research Committee of the Faculty of Veterinary Medicine, Kasetsart University, Bangkok, Thailand (Approval code: ACKU66-VET-075). The health status of these experimental animals was regularly monitored throughout the experiment. During the study, the crocodiles were housed in cement ponds at Wongveerakit Farm, in Kanchanaburi province, Thailand. Each pond had a surface area of 50 m^2^ and a pool depth of 25 cm. An environmental temperature range of 27–30 °C was registered during the experiment. The animals had not received any treatment for at least 1 month before the start of this study.

### 2.2. Drugs and Chemicals

The standard TA for calibration (purity > 98%) was purchased from Sigma Chemical Co. (St. Louis, MO, USA). Phenylbutazone (PBZ; purity > 99%) was used as the internal standard and was obtained from LGC Standards (Teddington, UK), while 4% TA (Tolfedine^®^; Vetoquinol; Lure, France) solution for injection was used for drug administration to the crocodiles. Purified water was produced using the Milli-Q water purification system from Millipore, Inc. (Bedford, MA, USA). Other reagents and chemicals of analytical grade were purchased from Sigma Chemical Co. (St. Louis, MO, USA).

### 2.3. Experimental Design

The study involved 15 freshwater crocodiles that were weighed and randomly divided into three groups of five animals. TA was administered at 2 mg/kg b.w., via either IM or IV, or at 4 mg/kg b.w. via IM in a parallel study design. For IM dosing, the injection site was at the left biceps and was delivered using a 1.5-inch needle. The IV injection was given at the post-occipital sinus with a 22-gauge, 1-inch needle. Blood samples (1.5 mL) for the pharmacokinetic analyses were collected from the tail vein of each animal at the various times: 0, 5, 15, 30 min, and 1, 2, 4, 8, 10, 24, 48, 72, 96, 120, 144, and 168 h post-dosing. Then, blood samples were centrifuged at 1986× *g* for 15 min to separate the plasma, after which the plasma was harvested and immediately stored at −20 °C for 2 weeks before analysis.

### 2.4. Analysis of Tolfenamic Acid Concentration in Plasma

The TA extraction method has been described by Raweewan et al. and Turk et al. [12,17]. Briefly, plasma samples (200 µL) were spiked with 20 µL of phenylbutazone standard solution (5 μg/mL) and then diluted with 200 µL of acetonitrile. The mixtures were vortex-mixed for 1 min and sonicated for 10 min. Afterward, samples were centrifuged at 17,500× *g* for 10 min. The organic layer was collected and passed through a 0.22 μm nylon filter. A 25 µL sample of supernatant was subjected to high-performance liquid chromatography (HPLC).

Plasma concentrations of TA were determined using an Agilent 1260 series system (Agilent Technologies; Santa Clara, CA, USA) consisting of a quaternary solvent delivery system with dual pumps, an autosampler, a column oven, and an ultraviolet detector set at a wavelength of 289 nm. The column was a reverse-phase, Hypersil^TM^ BDS C_18_ column along with a length of 150 mm × 4.6 mm inner diameter, 5 μm particle size (Thermo Fisher Scientific; Vilnius, Lithuania) and a C_18_ guard column (4.6 mm× 12.5 mm, 5 µm particle size; (ZORBAX Eclipse; Agilent Technologies; Santa Clara, CA, USA). The analytical C_18_ column was maintained at 35 °C. The isocratic mobile phase of the TA analysis contained 0.1% triethylamine in a Milli-Q-to-acetonitrile ratio of 75:25 *v*/*v* and delivered at a flow rate of 0.4 mL/min. The final solution pH was 3.0. The retention times for the TA and PBZ were approximately 8.7 min and 7.2 min, respectively.

### 2.5. Analytical Method Validation

Method validation was performed according to EMA [21] guidelines. The calibration standard of blank crocodile plasma was spiked with TA working standard solution to achieve final concentrations of 0.15, 0.25, 0.5, 1, 2.5, 5, and 10 μg/mL. The correlation coefficient (R^2^) value of the TA calibration curve was 0.999, confirming a strong correlation between the calibration curve and the experimental data. The validated limit of detection and quantification were 0.05 and 0.15 μg/mL, respectively. To assess recovery, the inter- and intra-day precision, and accuracy of the method, quality control samples at low (0.5 μg/mL), medium (5 μg/mL), and high (10 μg/mL) concentrations were prepared in five replicates within 5 days. The inter- and intra-day levels of precision were ≤7.12% and 6.15%, respectively. Accuracy was in the range of 87–111%.

### 2.6. Plasma Protein Binding Assay

Ultracentrifugation (Optima^TM^ Max-XP; Beckman Coulter, Inc.; Indianapolis, IN, USA) was used to assess plasma protein binding, as described by Howard et al. and Raweewan et al. [16,22]. Freshwater crocodile plasma free of TA was spiked with TA to create final concentrations in the range 0.5–10 μg/mL. Then, the prepared samples were transferred to 2 mL centrifugation tubes and centrifuged at 543,000× *g* for 2.5 h. The TA concentrations in the plasma and the resulting ultrafiltrate were determined using HPLC, as previously described. The percentage of plasma protein binding was calculated using the equation:

Protein binding (%) = Total concentration − Ultrafiltrate concentration/Total concentration (×100).

### 2.7. Pharmacokinetic Analysis

The average plasma concentration of the TA in the freshwater crocodiles was analyzed using a non-compartmental approach (PKanalix^TM^ R1;2023; Lixoft Software, France). The bioavailability (F) for the IM route was calculated using the following equation:

F (%) = (AUC_extravascular_/AUC_intravenous_) × (Dose_intravenous_/Dose_extravascular_) × 100.

### 2.8. Statistical Analysis

The plasma concentration-time data of TA and the values of the pharmacokinetic parameters were expressed as geometric mean values, except for the time of concentration to reach its peak (T_max_), which was presented as a median value. T_max_ was analyzed using the non-parametric Kruskal–Wallis test. One-way ANOVA was used to compare other pharmacokinetic parameters, with the Tukey post hoc test applied to assess differences between groups. Data analysis was conducted using the SPSS 27.0 software program (IBM Corp.; Armonk, NY, USA). Significance was tested at *p* < 0.05.

## 3. Results

The freshwater crocodiles remained in good general health and condition during the entire experimental period. There were no detected changes in behavior, activity, or appetite based on visual examination after the IV and IM drug administration events.

**Figure 1:**
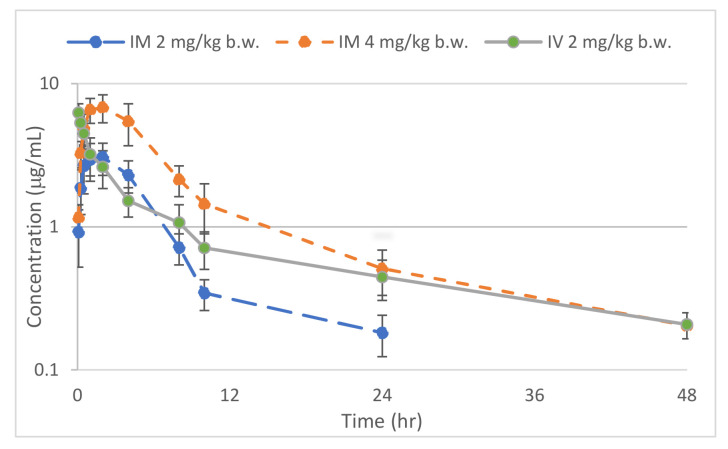
Semi-log average (±SD) plasma concentration versus time curve of tolfenamic acid after a single administration via intravenous (IV) at 2 mg/kg b.w. or intramuscular (IM) at 2 or 4 mg/kg b.w. in freshwater crocodiles (n = 5 per group), where error bars represent SD.

Semi-logarithmic plasma concentration-time curves of TA after administration as a single dose of 2 mg/kg b.w. IV, 2 mg/kg b.w. IM and 4 mg/kg b.w. IM routes were plotted and are shown in Figure 1. Data for the pharmacokinetic parameters determined following the IV and IM routes of administration of the TA are summarized in Table 1. The plasma concentration-time curves of TA (mean ± SD) were detected up to 24 h following IM administration at 2 mg/kg b.w. and up to 48 h after IM at 4 mg/kg b.w. and IV at 2 mg//kg b.w. administration. The maximum concentration (C_max_) values at the doses of 2 mg/kg b.w. and 4 mg/kg b.w. were 3.03 and 6.83 μg/mL, respectively. There were significant differences between the two doses of IM administrations and the IV routes of administration based on AUC_0–∞_, AUC_last_, MRT, t_½λz_, and C_max_. The average ± SD plasma protein binding for TA in this study was 26.15 ± 4.93%.

## 4. Discussion

To find the most effective yet safest dose of NSAIDs, it is crucial to understand how each active ingredient in this class of medications is absorbed, distributed, metabolized, and eliminated in each animal species. This study was the first to publish findings on the pharmacokinetics of TA administration in freshwater crocodiles. These results might be beneficial for managing pain and inflammation in these animals, whether they are raised on farms or housed in zoos or conservation centers.

The average ± SD plasma protein binding for TA in this study was 26.15 ± 4.93%, closely aligning with the reported values of 19.43% in green sea turtles [16] and 31.39% in hawksbill turtles [17]. However, it was notably lower than the 99.48% reported for rainbow trout [20]. This may have been a result of the comparatively lower levels of blood albumins in freshwater crocodiles (especially in reptiles as opposed to mammals), which may have influenced drug binding [23].

Following the IV administration in the freshwater crocodiles at the 2 mg/kg dose, the total Cl was 50.04 mL/h/kg, which exceeded the levels reported in turtles (1–30 mL/h/kg) [15,16,17,20], but was lower than those reported in birds (150 mL/h/kg) [24] and mammals (179–300 mL/h/kg) [11,14,15]. In general, reptiles have a lower metabolic rate than many other animals. As a result, most NSAIDs are cleared more slowly than in mammals. In the current study, the Vd of the TA was 1.58 L/kg, which was higher than has been reported in sheep, trout, green turtles, hawksbill turtles and red-eared sliders [13,15,16,17,20]. In general, NSAIDs are strongly bound to plasma proteins; therefore, their volumes of distribution are low. The binding ratio of TA-to-plasma proteins in the current study was low (26.15%) compared to other mammalian species. The reason for the different Vd values for the TA between species may have been due to anatomical and physiological differences and changes in the binding ratio of the drug to plasma proteins.

Notably, the observed C_max_ of TA in the freshwater crocodiles at a 2 mg/kg b.w. dose (3.03 μg/mL) was lower than the values reported in pharmacokinetic studies conducted in rainbow trout (8.82 μg/mL), red-eared sliders (6.87μg/mL), geese (4.89 μg/mL), Japanese quails (13.49 μg/mL), and ducks (4.59 μg/mL) at the same dose [12,15,18,19,20]. However, this value was higher than those reported in goats (1.77 μg/mL and 1.635 μg/mL) [11,25]. At the higher dose of 4 mg/kg b.w., the C_max_ value was 6.83 μg/mL, which was similar to that recorded in female rats (7.11 µg/mL), but lower than that obtained in green turtles (55.69 µg/mL) and hawksbill turtles (89.33 µg/mL) [14,16,17]. The difference in C_max_ across these species may be attributed to variations in metabolic rate, absorption efficiency, or differences in the area of administration. Furthermore, the T_max_ values were identical after IM administration of TA at doses of 2 and 4 mg/kg, indicating that the rate of absorption was not dose-dependent.

The absolute bioavailability levels of TA after the 2 mg/kg b.w. and 4 mg/kg b.w. doses following IM administration in the freshwater crocodiles were 71.01% and 92.63%, respectively. In other studies, IM bioavailability of TA was reported in goats (66.46% [11], green sea turtles (72.02% [16], rainbow trout (85.87% [20], ducks (93.62% [18], hawksbill turtles (94.46% [17], and red-eared slider turtles (110.28% [15]. These results suggested that the drug was efficiently absorbed from the site of injection, had optimal drug solubility, and had been formulated to enhance its systemic availability.

After IV administration of 2 mg/kg b.w. and IM administration at 2 mg/kg b.w. and 4 mg/kg b.w., the t_1/2ʎ_ values were 21.89 h, 17.14 h, and 13.57 h, respectively. Increasing the dose may have resulted in a shorter elimination half-life due to the NSAIDs exerting their effects by inhibiting the synthesis of PGs. Higher doses of NSAIDs reduced blood flow to the kidneys by inhibiting the synthesis of prostaglandins, thereby having a potentially adverse impact on renal function [26,27]. The prolonged half-life of TA, which varied depending on the dosage, might lead to decreased clearance due to reduced renal blood flow. The results after IV administration were consistent with findings in the red-eared slider (17.55–20.39 h; [15], while being notably shorter than those reported in other turtle species (32.76–38.93 h; [16,17] and longer than reported in mammals (1.60–3.51 h; [11,12,13,14,28], fish (6.75 h; [20] and birds (1.95 h; [24]. The small animal sample size used in the present study might also have had an impact on the half-life values. Further studies with larger animal sample sizes would be warranted to clarify this issue. While pointing to differences in pharmacokinetic behavior after IM administration, t_1/2ʎz_ was faster than in other reptile species (22.49–41.09 h; [15,17]. However, relatively shorter t_1/2ʎz_ values for TA have been reported in goats (2.97 h; [11] and rainbow trout (6.75 h; [20] and shorter than those reported in birds (1.51 h; [19] and mammals (2.97 h). There was similar with the MRT, with the mean MRT values being 13.44 h for the 2 mg/kg dose and 12.68 h for the a 4 mg/kg dose following IM administration, and 23.52 h for IV administration for the 2 mg/kg dose. These results were comparable to those for the red-eared slider (20.39; IV; Corum et al., 2019) and exceeded those reported in mammals (1.30–1.59 h; IV and 3.89–8.06 h; IM; [11,13,28], in ducks (2.12 h; IV and 3.04 h; IM; [18], but were shorter than the values reported in other turtle species (37.10–53.84 h IV and 28.06–61.99 h IM; [16,17].

In some parts of the world, farmed crocodiles are raised for both their skin and meat production. With the long half-life of tolfenamic acid observed in this study, it is necessary to carefully consider the implications for the withdrawal period before the meat or skin of treated crocodiles can be safely marketed for consumption or product use. The extended half-life could result in the need for prolonged withdrawal time to ensure that the drug has been fully cleared from the animal’s system, particularly when the meat is intended for human consumption. These findings underscore the critical need for further pharmacodynamic studies to establish safe and practical dosing of NSAIDs in food-producing animals while protecting both animal welfare and consumer health. 

In mammalian species, TA is extensively metabolized in the liver, excreted via bile and urine, and re-enters circulation through the enterohepatic cycle [29]. Variations in the t_1/2λz_ and Cl vlaues of TA across species may be influenced by species-specific characteristics, of which the most influential is temperature [21]. Lower temperatures reduce oxygen consumption and tissue metabolic requirements may be attributed to their lower metabolic rate, leading to slow metabolism and prolonged elimination of TA in freshwater crocodiles. These findings emphasize how physiological differences between species can greatly influence the pharmacokinetics of TA.

Earlier studies reported the IC_50_ concentrations in plasma required to exudate prostaglantin E2 and thromboxane B2 in mammals were in the ranges of 0.07–0.23 µg/mL and 0.26–1.3 µg/mL, respectively [25,30,31]. These data have been used to estimate the effectiveness of TA between species. However, such speculation in crocodiles should be discouraged for two main reasons. The first is that the animal species investigated in the present study might have a pain threshold very different from those known in mammalian species. Secondly, the difference in plasma protein binding detected between mammals and crocodiles makes the TA plasma concentration three times more available in the bio-phase in crocodiles. Consequently, a specific study designed to investigate the pharmacodynamics of TA would be the only way to evaluate its effectiveness in this animal species and avoid speculation regarding adverse effects.

## 5. Conclusions

The study revealed no adverse effects associated with the administration of a single dose of TA after IM (2 or 4 mg/kg) or IV (2 mg/kg) routes in freshwater crocodiles. The pharmacokinetic analysis demonstrated prolonged values of t_1/2λ_, low clearance, and a high volume of distribution. Despite these findings, further research is essential to establish an optimal dosage regimen, ensuring the drug’s safety, efficacy, and therapeutic applicability under varying clinical conditions in this species. The potential concern of prolonged half-life in this study underscores the critical need for further pharmacodynamic studies and establish safe and practical dosing of NSAIDs in food-producing animals while protecting both animal welfare and consumer health.

## Figures and Tables

**Table 1:** Geometric mean values evaluated for pharmacokinetic parameters of freshwater crocodiles following a single dose of tolfenamic acid administered intravenously (2 mg/kg b.w.), or intramuscularly (2 mg/kg b.w. or 4 mg/kg b.w.).

Parameter	Unit	Intravenous (IV)			Intramuscular (IM)					
		2 mg/kg (n = 5)			2 mg/kg (n = 5)			4 mg/kg (n = 5)		
		GM	Max	Min	GM	Max	Min	GM	Max	Min
λ_z_	1/h	0.003	0.004	0.002	0.003	0.004	0.003	0.005	0.006	0.004
t_1/2λz_	h	21.89	25.07	17.28	17.74 ^a^	21.03	15.61	13.57 ^a,b^	15.37	11.12
T_max_ ^§^	h	–	–	–	2.0	2.0	0.5	2.0	2.0	1.0
C_max_	μg/mL	–	–	–	3.03	4.38	1.89	6.83 ^b^	9.56	4.92
AUC_0–last_	h × μg/mL	33.47	44.09	22.85	23.77 ^a^	34.08	17.26	62.01 ^a,b^	86.45	40.09
AUC_(0–∞)_	h × μg/mL	39.96	52.77	27.91	25.70 ^a^	35.88	18.76	65.89 ^a,b^	89.82	42.27
Vd	L/kg	1.58	2.59	1.11	–	–	–	–	–	–
Cl	mL/h/kg	50.04	71.65	37.90	–	–	–	–	–	–
MRT_0–inf_	h	23.52	26.42	21.41	13.44 ^a^	15.45	11.42	12.68 ^a^	15.27	10.62
F	%				71.01			92.63		

Abbreviation: C_max_ = maximum concentration, T_max_ = time of peak concentration, λ_z_ = elimination rate constant, t_1/2λ_ =elimination half-life, AUC_0–last_ = area under the curve from 0 to the last point of drug quantification, AUC_0–∞_= area under the curve from 0 h to infinity and MRT_0–inf_ = mean residence time from 0 to infinity. F = absolute bioavailability, Vd = volume of distribution, Cl = clearance. ^a^ Significantly different compared to the intravenous route (*p* < 0.05). ^b^ Significantly different between intramuscular routes (*p* < 0.05). ^§^ Median value.

## Data Availability

Dataset available on request from the authors.
